# Generation and Assessment of Soybean (*Glycine max* (L.) Merr.) Hybrids for High-Efficiency *Agrobacterium*-Mediated Transformation

**DOI:** 10.3390/life14121649

**Published:** 2024-12-12

**Authors:** Muhammad Waqar Khan, Aaqib Shaheen, Xuebin Zhang, Junli Zhang, Yaser Hassan Dewir, Katalin Magyar-Tábori

**Affiliations:** 1 State Key Laboratory of Crop Stress Adaptation and Improvement, Henan Joint International Laboratory for Crop Multi-Omics Research, School of Life Sciences, Henan University, Jinming Road, Kaifeng 475004, China; xuebinzhang@henu.edu.cn (X.Z.); zhangjunli0532@163.com (J.Z.); 2 State Key Laboratory of Crop Stress Adaptation and Improvement, College of Agriculture, Henan University, Kaifeng 475004, China; aaqibtoshaheen@gmail.com; 3 Plant Production Department, College of Food and Agriculture Sciences, King Saud University, Riyadh 11451, Saudi Arabia; ydewir@ksu.edu.sa; 4 Research Institute of Nyíregyháza, Institutes for Agricultural Research and Educational Farm (IAREF), University of Debrecen, P.O. Box 12, 4400 Nyíregyháza, Hungary; mtaborik@agr.unideb.hu

**Keywords:** soybean breeding, transformation, flavonoids, susceptibility, UPLC-MS/MS

## Abstract

The *Agrobacterium*-mediated technique is widely employed for soybean transformation, but the efficiency of this method is still relatively modest, in which multiple factors are involved. Numerous chemical and physiological cues from host plants are needed for *A. tumefaciens* attraction and subsequent T-DNA integration into the plant genome. Susceptible genotypes may permit this attachment and integration, and the agronomically superior genotypes with susceptibility to *A. tumefaciens* would play an important role in increasing transformation efficiency. In this study, we aimed to elevate the *Agrobacterium*-mediated transformation efficiency of soybean by integrating susceptibility alleles from William82 and flavonoids accumulating alleles from LX genotypes in the same soybean line. The crossing was made between LX (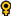) and William82 (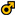) soybean by hand pollination. Expectedly, the resulting hybrid soybean progenies inherited susceptibility traits and high flavonoid contents (i.e., genistein, genistin, apigenin, naringenin, quercetin, and cinnamic acid) essential for potential plant–pathogen interaction. Furthermore, the progenies and susceptible William82 soybean were subjected to transformation using *A. tumefaciens* (GV3101) harboring the GmUbi-3XFlag-35S-GFP and reassembled GmUbi3XFlag-35S-GFP: GUS vectors during separate events. Important transformation-related traits like shoot induction and shoot regeneration ability were also significantly improved in progenies. The progenies designated as ZX-3 exhibited superiority over the William82 parental line in all three traits, i.e., shoot induction, regeneration, and *Agrobacterium*-mediated transformation. The transient transformation efficiency of the ZX-16 line was remarkably higher when half-cotyledon explants were wounded and transformed with *A. tumefaciens* harboring GUS assembly vector and then co-cultivated on MS medium supplemented with 2 mg/L spermidine, 0.3 g/L GA3, 0.3 mg/L kinetin, and 1.3 mg/L 6-benzylaminopurine. In addition, the shoot elongation was also higher than that of William82 after two weeks of culture on the shoot induction medium. The newly generated soybeans have the potential to serve as a valuable source for high transgene production and represent a promising avenue for future soybean varietal development.

## 1. Introduction

Conducting transformation breeding in susceptible genotypes is a challenge due to the inherent recalcitrant nature of soybean (*Glycine max* (L.) Merr., Fabaceae) [1]. Only 5% of the plant susceptibility traits to *Agrobacterium* are environmentally dependent, whereas genes control about 95% of them. The trait of susceptibility is controlled by additive genes in *Brassica oleracea* and could be transformed into resistant genotypes with high heritability [2]. However, the heritability of this trait is controlled by a single locus in *Arabidopsis,* which proved to be quantitative [3] and has also been confirmed in some other plants [4]. This trait exhibits a high likelihood of heritability in the soybean. The susceptibility observed in resistant soybean genotypes can be attributed to the introgression of susceptibility-related traits via conventional breeding methods [5]. Numerous quantitative traits Loci are crucial to plant–pathogen interactions and T-strand trafficking. The host cell protein likely aids in the nuclear import of the T-complex. The VIP1 protein enhances Arabidopsis susceptibility to *A. tumefaciens* for both transient and stable transformation [6]. However, different traits cause different susceptibilities in different genotypes. Many soybean genotypes are resistant to *Agrobacterium* infection, even though they have a variety of desirable traits, including high levels of chemoattractants and *vir* gene inducers (polyphenolics). Plant-derived signaling, seen in a range of plant genotypes, speeds up the *Agrobacterium* transformation [7]. An extensive survey of the susceptible plant families to *Agrobacterium* revealed that these families accumulate high levels of polyphenolic compounds [8]. The phenolic compounds produced by injured plant tissues initiate the activation of two-component systems, namely *Vir*A and *Vir*G, within the *Agrobacterium*. Moreover, the addition of some sugar and acid facilitates the transfer of T-DNA from bacteria to the host genome [9]. A large number of flavonoids have also been investigated as crucial players in the *Rhizobium* symbiosis. The role of flavonoids in increasing host range susceptibility has been mostly taken into consideration. A small quantity (1 µM to 0.1 nM) of flavonoids acts as chemoattractant and node gene activators [10]. Flavonoids like apigenin 7-glucoside, myricotin, rutin, and cinnamic acid were found to be more effective in the natural *Agrobacterium* infection process as compared to acetosyringone in microalgae and tobacco [11]. These flavonoids are released by the host and carry out a variety of actions, notably enabling the T-strand to cross the cell membrane and nuclear membrane in order to enter the cytosol and, finally, the nucleoplasm. Despite the important role that flavonoids play in the interaction between *Agrobacterium* and plants, it is difficult to transfer flavonoid loci to sensitive soybean genotypes [12]. This is due to the complex regulation of flavonoid accumulation, which involves more than 138 quantitative trait loci (QTLs) in some plants [13].

The accumulation of characteristics that facilitate *Agrobacterium* infection and the integration of its T-DNA into the plant genome is a crucial element of our work to attain high transformation efficiency in the soybean. The incorporation of susceptibility and elevated flavonoid-related characteristics into a single genotype would be particularly advantageous for efficient *Agrobacterium*-mediated transformation. To achieve our ambitious transformation objective while ensuring agronomic excellence, our research aimed to transfer susceptibility-related traits to a high flavonoid-accumulating resistant soybean cultivar. We used the high flavonoid accumulating soybean landrace LX (Lin Xian Xiao Huang Dou) as the female parent and the susceptible variety William82 (Wm82) as the male parent for hybridization. The resulting progeny were assessed for high efficacy in *Agrobacterium*-mediated transformation and other agronomically significant features. We assessed these effects using the reassembled vectors containing GUS and GFP reporters [14]. The basic characteristics that confer high susceptibility to soybean, like short maturity, high flavonoids, and cinnamic acid, were also assessed. The number of regenerated shoots on explant and the shoot regeneration capabilities of progenies were investigated.

## 2. Materials and Methods

### 2.1. Selection and Cultivation of Parental Lines Under Controlled Conditions

For the development of the cross-population, we selected LX (Chinese-origin landrace with high flavonoid content) [15] and Wm82 (a susceptible genotype). LX was selected as a female, and Wm82 was selected as a male, which is an early-maturity soybean variety mostly used for efficient *Agrobacterium* transformation. To produce cross population, healthy seeds from each parent were selected and grown under controlled conditions inside the growth chamber (24 °C, 70% RH, 14/10 light/dark photoperiod). More than 100 seeds from each parental line were sown in pots containing a mixture of vermiculite: peat (1:1; *v*/*v*). The seeds were germinated and speed-bred to the R1 stage, as described by Jähne et al. [16]. The plants were regularly watered with water-soluble nitrogen fertilizers. Healthy plants were selected for cross-pollination.

### 2.2. Hand Pollination of Flowers

The hand pollination was carried out inside the growth chamber in April 2020. The pollens from William’s 82 were collected in tubes a day before pollination. The emasculation of female flowers was carried out before the anthesis. The stigma of LX flowers was uncovered with forceps by carefully removing five sepal lobes and the parted corolla and covered with bags during pollination. A magnifying lens was used to ensure successful pollination and protect the stigma from damage. The pollens were shed on the stigma, as described by Kim et al. [17]. More than 80 crosses were made in the female parental lines. Successful crosses were obtained with a 3.3% crossing efficiency. The seeds were harvested in June 2020, within 70 days. The F1 progenies were raised under environmental conditions as described. In each population cycle, progeny exhibiting increased anthocyanin coloration and early maturity were selected and raised for subsequent generations. Complete homozygosity was attained in the F5 generation. However, the selection was further made for two more subsequent generations to mitigate inbreeding depression within the population. The F7 generation was achieved in two and a half years.

### 2.3. Extraction of Flavonoids

The extraction of flavonoids was carried out through a modified protocol as described by Rodríguez De Luna et al. [18]. Dried powder from each sample (0.1 g) was placed in a 10 mL tube, and a total volume of 5 mL of aqueous ethanol was added to the powder. The mixture was thoroughly mixed by using an Adolph mixer (Germany) for 10 min. The tubes containing the mixture were incubated at room temperature for 12 h. The supernatant from each sample was filtered into a separate tube. The solid masses were re-suspended with 2 mL of fresh 96% ethanol. The extract was homogenized for 20 min and then filtered. The filtrate of each sample was desiccated in an Eppendorf desiccator for 8 h. The dried masses were then re-suspended in a small volume (300 µL) of 96% ethanol. The mixtures were vigorously agitated for 5 min. Before loading into HPLC, the extract was filtered using a 0.2 µm syringe filter. From each sample, 200 µL of the extract was loaded into an HPLC vial for quantification of different flavonoids.

### 2.4. Quantification of Flavonoids and Cinnamic Acid

Five flavonoids (apigenin, quercetin, genistein, naringenin, luteolin, and cinnamic acid) HPLC-grade standards were obtained from a lab-maintained chemical repository. The standard solution of 1 mg/mL was prepared using dimethyl sulfoxide (DMSO). The standards were diluted further for HPLC-QqQ-MS/MS analysis. A calibration curve was obtained, and a correlation coefficient of 0.99% was obtained for all the standard solutions. The HPLC-QqQ-MS/MS was run under normal calibration in the Xevo TQ-S micro. Positive electrospray ionization (ESI) was used to acquire ion peak while running the machine in multiple reaction monitoring mode (MRM). The mobile phase “A” was double distilled water (ddH_2_O) with 0.01% formic acid (HPLC grade), and “B” was 80% aqueous methanol (HPLC grade). The elution gradient of 0.4 mL solution was used in the range of 0 min at 5% B, 0.5 min at 5% B, 2.5 min at 20% B, 13 min at 80% B, 15 min at 95% B, 16 min at 95% B, and 18 min at 5% B. The injection volume was 1 µL. A color spectrum column (AcquityUPLC cortecs T3; 2.1 × 100 mm, 1.6a; P/N: 186008499; Water^TM^, TX, USA) was used for the quantification of flavonoids. The column and sample temperatures were 35 °C and 8 °C, respectively. The desolvation gas temperature was 500 °C, and the desolvent gas flow rate was 800 L/h. All the samples had three biological replicates. The quantities of each sample were obtained in µg/mL. The experiment was repeated three times for further validation.

### 2.5. Shoot Regeneration and Multiple Shoot Induction

Shoot regeneration is an important step in soybean transformation. The new soybean lines were evaluated for their shoot regeneration capabilities. More than four hundred half-cotyledon explants were evaluated for shoot regeneration, shoot induction, and transformation from each line, along with Wm82 as a control. The seeds were cultured on a quarter-strength Murashige-Skoog (MS) medium [19] with B5 vitamins for 18 h. The plumules from half cotyledons were removed. The explants were cultured onto full-strength MS medium with B5 vitamins, 2% sucrose, and 0.7% agar, and the pH of the medium was adjusted to 5.7 with (1.5 g/L) MES. We used our lab-established combination of plant growth regulators (PGR) in MS medium for rapid shoot induction (spermidine 2.5 mg/L (cat# S8030), 6-Benzylaminopurine (BAP) 1.3 mg/L (IB0100), GA_3_ 0.3 mg/L (cat# G8910), kinetin 0.3 mg/L (cat# K8011), carbenicillin 200 mg/L, and cefotaxime 150 mg/L (purchased from Solarbio Life Sciences; Peking, China). The number of shoots was counted on each regenerated explant for each soybean line. After 14 days, regeneration efficiency was calculated by dividing the number of regenerated explants by the total number of explants.

### 2.6. Construction of Vectors and Explant Preparation

Soybean cotyledon explants were infected with *Agrobacterium tumefaciens* (GV3101) harboring the GmUbi-3XFlag-35S: GFP and modified GmUbi-3XFlag-35S: GUS/GFP vectors to assess the transformation efficiency. For the construction of the GUS expression vector, computer simulation was performed in Snapgene software version 6.0.2 by using GmUbi-3XFlag-GFP as the destination vector. The GUS fragment was amplified from the pCambia1300 vector by using pairs of overlapping primers: the forward primer GACTCGACAGTCTAGAATGGGTTTACGTCCTGTAGAAACC and the reverse primer TCCTTATAGTCCATGGTACCTCATTGTTTGCCTCCCTGCTGC. The vector was linearized with AscI and XbaI restriction endonucleases. Digestion of the vector was carried out in a 50 mL reaction volume containing 1 µg plasmid, 1 µL each of enzyme, 5 µL quick cut buffer, and ddH_2_O. The reaction mix was incubated at 35 °C for 2 h. The digestion of the vector was confirmed in 0.8% agarose gel. The digested vector was purified from the gel and allowed to ligate with the GUS fragment containing a complementary overhang. Ligation was performed using a one-step DNA cloning kit (Novoprotein; Abkang Trading Co., Ltd., Shanghai, China) following the user’s manual. The ligated vector was transformed into a competent bacterial cell by the heat-shock method. The selection of true colonies was made through colony PCR, and then the complete GUS gene was identified through Sanger sequencing. The positive colonies were cultured in LB liquid media (Tryptone, 1 g/100 mL, yeast extract, 1 g/100 mL, and 0.5 g/100 mL NaCl) [20] with selective antibiotics overnight. The vector was extracted from the bacterial colonies. The vector was then transformed into *Agrobacterium* GV3101 by the freeze-thaw method. The positive *A. tumefaciens* was cultured in YEP media with selective antibiotics overnight. The overnight culture was then stored with 30% glycerol (1:1) in a −80 °C freezer for future use. Prior to inoculation, freshly harvested seeds from soybean lines were sterilized in 70% ethanol (5 min) and 4% sodium hypochlorite (14 min). The seeds were washed with distilled water multiple times. The seeds were allowed to imbibe for 18 h on a germination medium containing quarter-strength MS with vitamins, 1% sucrose, and 0.7% agar with adjusted pH of 5.7. The imbibed seeds were sliced in half. The half-cotyledon explants were inoculated with *Agrobacterium* harboring the GmUbi-3xFlag-35S: GUS/GFP binary vector. The inoculation media consists of half-strength MS media with B5 vitamins, 2% sucrose, 250 mg/L L-cysteine, and 200 µM acetosyringone. The half-cotyledon explants were excised, and multiple cuts were made adjacent to the cotyledonary node. The wounded explants were placed in inoculated media for 30 min (OD600 = 0.7 ± 0.1). The explants were then transferred to sterile filter paper moistened with co-cultivation medium (half-strength MS with B5 vitamins, 2% sucrose, 250 mL L-cysteine, 200 µM acetosyringone, 0.7% agar, 0.3 mg/L GA_3_, 2 mg/L spermidine, and 1.3 mg/L 6-BA, pH 5.4) for four days. After co-cultivation, the explants were washed in half-strength MS liquid media containing 250 mg/L cefotaxime and 250 mg/L carbenicillin for 30 min. The explants were then transferred to a shoot induction medium as described for shoot regeneration.

### 2.7. Confirmation of Transgenesis Through GUS and GFP Signals

After two weeks of shoot regeneration and elongation, the cotyledon explants were shifted to fresh shoot induction medium (SIM) containing full-strength MS with B5 vitamins, MgCl_2_·6H_2_O 0.2 mg/L and agar 0.7%. Different growth regulators, 6-BA 1.3 mg/L (Solarbio IB0100), spermidine 2.5 mg/L (Solarbio cat# S8030), N6-(2-isopentenyl) adenine 2iP 0.4 mg/L (cat# B24576), kinetin 0.3 mg/L (Solarbio, cat# K8011), GA3 0.3 mg/L (Solarbio cat# G8910), cefotaxime 150 mg/L, carbenicillin and 200 mg/L (Solarbio) were added in small quantities. The 2-(N-Morpholino) ethane sulfonic acid monohydrate (MES) (Sigma Aldrich pcode 102461002) was added at 1.5 g/L to stabilize the pH at 5.7 as described by Khan et al. [21]. Putative transgenic lines were screened for transgenic events by using PCR, GFP fluorescence, and GUS histochemical assays. For T-DNA identification, total genomic DNA from each line was extracted. The PCR program was set to detect the T-DNA section in the soybean genome by using a pair of primers: forward ATTTTACAAATACAAATACATACTAAGGGTTTCT and reverse GCACCATCTTCTTCAAGGACG. The GFP fluorescence was detected in putatively transgenic soybean leaves using a confocal microscope (LSM980). For GUS expression analysis, the epicotyl of transformed explants was subjected to GUS histochemical analysis. The explants were incubated in GUS buffer (80 mM NaH_2_PO_4_ (pH 7.2), 10 mM Na_2_EDTA, 0.1% (v/v) Triton-X100, 2 mM K_4_Fe(CN)_6_, 2 mM K_3_Fe(CN)_6_, and 2 mM X-gluc) overnight at 37 °C as described by Jefferson et al. [22]. Similarly, all half-cotyledon explants were subjected to GUS analysis as described. The transformation efficiencies of progenies were compared with those of the susceptible parental line Wm82 by using statistical and mathematical methods. All the experiments were repeated three or more times.

### 2.8. Statistical Analysis

All experiments were set up in a completely randomized design, and the average values were taken from the mean of three replicates. The regeneration frequency, shoot induction rate, and transient transformation frequency were expressed as the mean ± standard deviation. Statistical differences among all the data were analyzed through a one-way ANOVA (Bartlett’s Test) at the 0.05 level of significance.

## 3. Results

### 3.1. Speed Breeding Under Controlled Conditions

**Figure 1:**
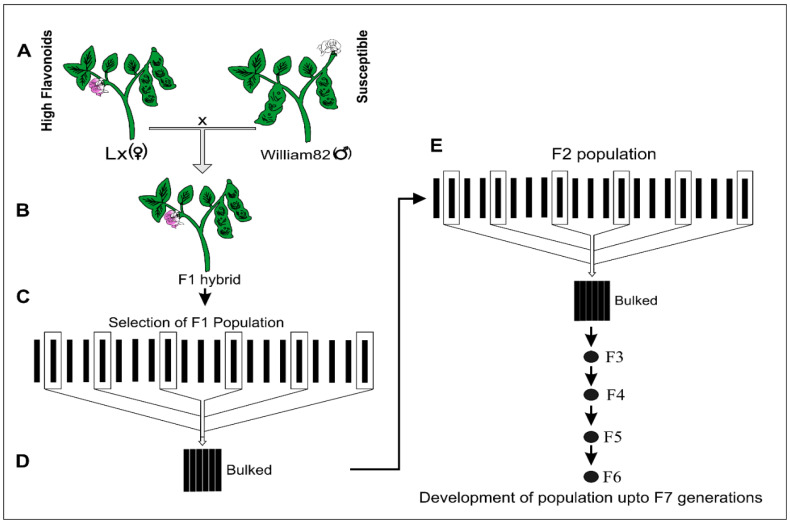
A diagram of soybean hybridization for susceptibility to *Agrobacterium* and production of agronomically superior soybean progenies. (**A**) LX (female) and Wm82 (male) were crossed by hand pollination. (**B**,**C**) The F1 hybrid population was further developed by selecting single lines from each population. The seeds from each line were bulked (**D**) and raised to the next generations (**E**).

The process of homozygous line attainment after successful cross-pollination was completed in 2.5 years inside the growth chamber under controlled conditions. The utilization of speed breeding has been recently employed in the soybean for varietal development [23]. We skipped the evaluation of progenies for transformation during F1 or F2 generations, as previously described [5], because the transfer of susceptibility traits to progenies remains normal; however, owing to frequency distribution, the stability of these traits can only be effective in homozygous lines. As a result, a homozygous line in the F7 generation was considered for further validation. A combination of breeding protocols was adopted to get superior genotypes within the shortest possible time. Vigorous plants were tagged as having (1) a high level of anthocyanin and short maturity and (2) a high level of anthocyanin and relatively long maturity during each generation. The inferior lines were eliminated prior to reaching maturity to avoid any contamination of the uniform lines. Among the 35 segregating populations in the F2 generation, only two progenies progressed to the F7 generation (Figure 1). The newly developed lines designated as ZX-16 exhibited high anthocyanin content and matured a week earlier (65 days) than the Wm82 parents, while ZX-3 was characterized as a late maturity line (75 days compared to Wm82’s 72 days). The flavonoid and cinnamic acid contents of these two lines were further assessed using UHPLC-QqQ-MS/MS analysis.

### 3.2. Elevation of Selected Flavonoids in New Soybean Progenies

**Figure 2:**
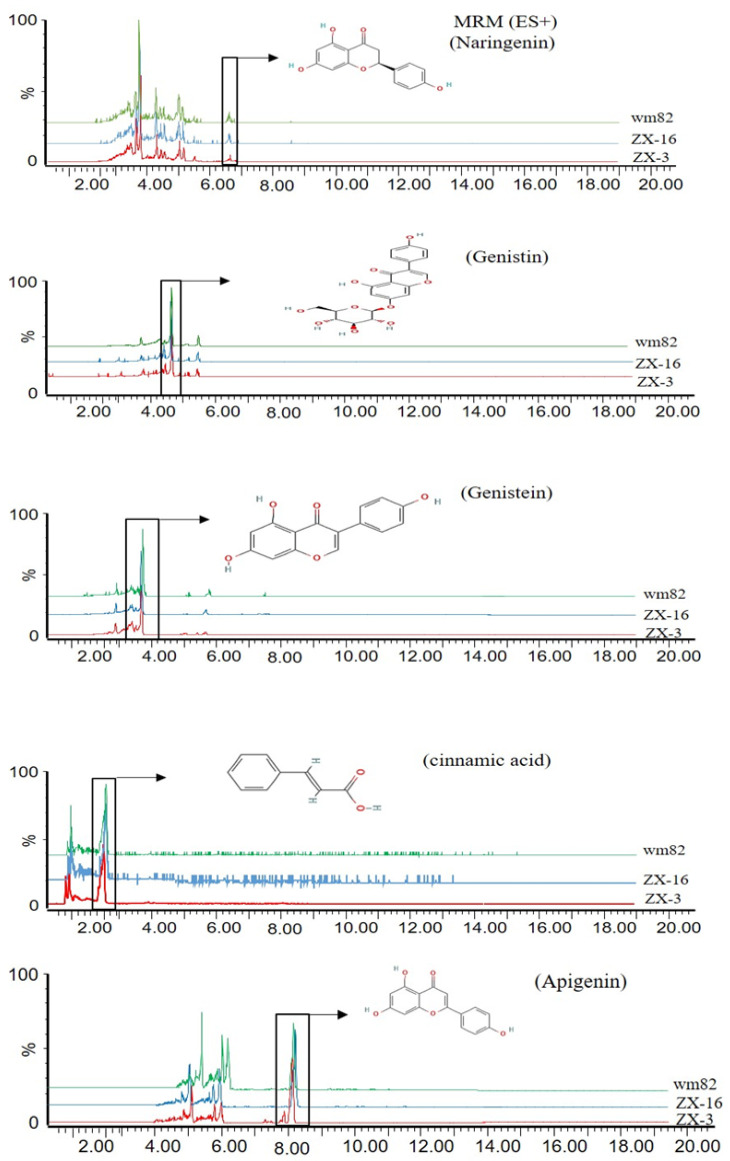
Extracted ion chromatogram (EIC) of different flavonoids obtained through UPLC-QqQ-MS/MS. The intensities of each chromatogram are given in percentages. Each picture shows the intensities of each compound in three soybean genotypes.

**Figure 3:**
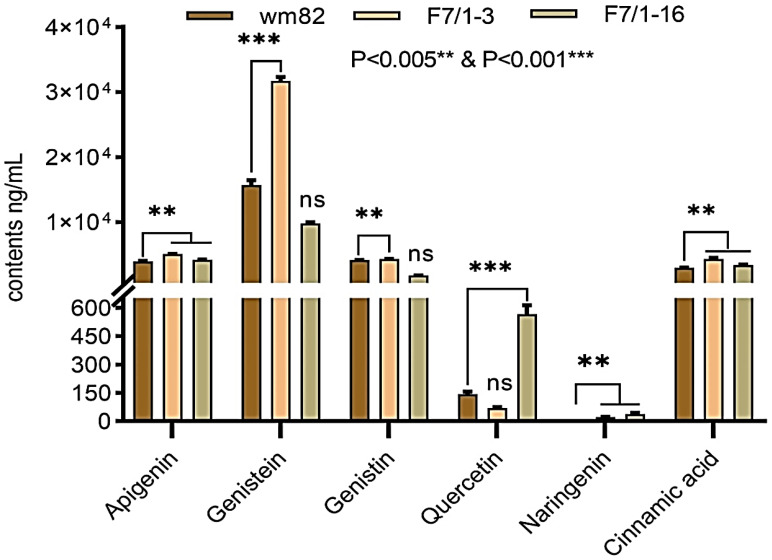
The quantification of flavonoids and cinnamic acid through UPLC-QqQ-MS/MS in two soybean progenies (F7/1-3 and F7/1-16), their susceptible parental line Wm82. Different flavonoid accumulation and cinnamic acid concentrations were assessed by comparing them with their standard solutions of known concentration. Statistical differences among all the data were analyzed through one-way ANOVA (Bartlett’s Test) at the 0.05 level of significance. However, the significance of individual compounds was analyzed through Multiple T-test statistics. Each line shows significance in high flavonoid accumulation at *p* < 0.01 ** and *p* < 0.001 ***. ns = non-significant.

The flavonoid compounds were extracted and quantified using UHPLC-QqQ-MS/MS. The quantity of each flavonoid was calculated by comparing them with a standard solution of known concentration. The UHPLC extracted ion chromatogram results indicated the variation in the accumulation of these flavonoid compounds in new soybean lines (Figure 2). Based on the Multiple T-test statistics, the abundance of apigenin, genistein, naringenin, and cinnamic acid was significantly higher (*p* < 0.01) in the ZX-3 line as compared to the Wm82 parents. Nevertheless, apigenin, naringenin, quercetin, and cinnamic acid were significantly abundant (*p* < 0.01) in the ZX-16 line as compared to Wm82. The comparison between hybrid lines showed a high abundance of four compounds. i.e., Apigenin, genistein, genistin, and cinnamic acid in the ZX-3 line, while two compounds, quercetin and naringenin, were abundant in the ZX-16 line. However, no detectable luteolin concentrations were found in any of the parental or hybrid lines after multiple attempts. The highest accumulation of quercetin was detected in the ZX-16 line; however, this compound was not detected in the female parental line (LX). These findings demonstrated that the quercetin biosynthesis allele is highly heritable and is unique to the Wm82 genome. More research is needed to clarify the transmission of this trait. The high accumulation of these compounds may lead to high-efficiency *Agrobacterium*-mediated transformation (Figure 3). To clarify this, we evaluated the transformation efficiency of these lines through *Agrobacterium-*mediated gene transfer.

### 3.3. Positive Response of Shoot Regeneration and Shoot Induction After Allele Segregation

**Figure 4:**
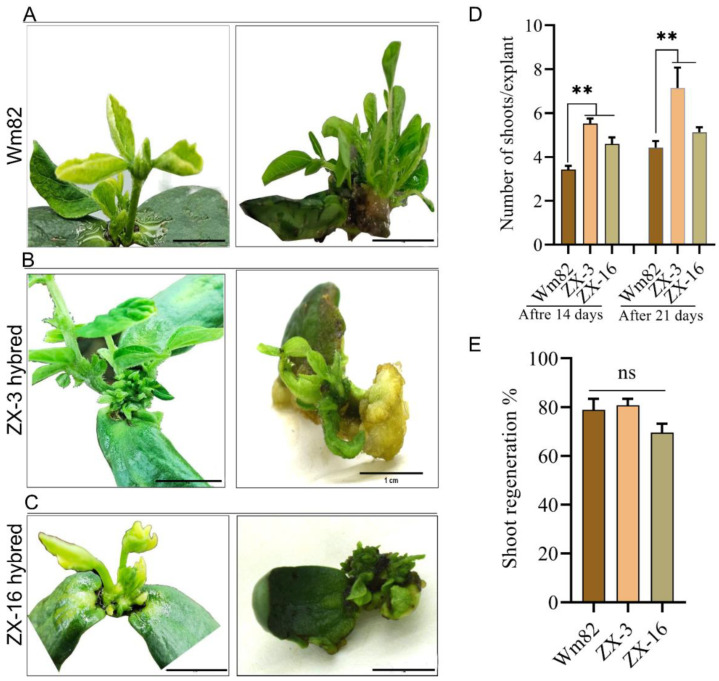
Evaluation of shoot induction and regeneration capabilities of hybrid lines on solid MS medium ((**A**–**C**), Scale bar = 1 cm). The performance of half-cotyledon and full-cotyledon explants of Wm82, ZX-3, and ZX-16 genotypes for their regeneration after 14 days of culture on solid MS medium or without solid medium. (**D**,**E**) The bar graph shows the number of shoots/explant and the average percentage of shoot regeneration in hybrid lines, respectively. ns and ** = non-significant and significant at *p* ≤ 0.01, respectively.

Prior to high-efficiency transformation, shoot induction and regeneration were evaluated. The shoot regeneration capabilities and number of newly induced shoots on explants were compared to their parental Wm82 line. For the shoot induction and regeneration, we used both solid MS medium and our newly developed solid medium-free protocol. The three genotypes were evaluated for shoot regeneration efficiency and the number of regenerated shoots (Figure 4A–C). A thorough evaluation was performed on 380 explants to assess the effectiveness of shoot induction and the resulting number of regenerated shoots across three different experiments. An average shoot induction capability of 80.7% was achieved in the ZX-3 line, leading 78.8% in Wm82, while the ZX-16 recorded the lowest 69.5% (Figure 4E). The shoot induction capability of the Wm82 parental line was comparable to that of the new hybrid lines. This response is favorable, as the Wm82 parental line also demonstrated exceptional characteristics associated with shoot induction. The new hybrid line ZX-3 showed the best performance over this trait. The number of regenerated shoots was counted after four weeks of explant culture on a solid medium. A significantly large number of elongated shoots were found in the ZX-3 and ZX-16 lines, compared to the Wm82 parental line, both two and three weeks after being transferred to a fresh medium (Figure 4D). Comparable outcomes were observed when shoot development was stimulated in explants utilizing our newly established solid media-free approach. The newly produced soybean lines have acquired specific advantageous qualities from the Wm82 line, including shoot induction and regeneration capabilities. Furthermore, these lines exhibited a significant increase in flavonoids and cinnamic acid, as previously noted in the LX parental line. The attainment of high-efficiency *Agrobacterium* transformation in one or both of the newly created lines constituted a unique combination. Consequently, we evaluated these lines for *Agrobacterium*-mediated transformation via cotyledon explant inoculation and culture.

### 3.4. Identification of the Inheritance of High Susceptibility to Agrobacterium in New Soybean Progeny Lines

**Figure 5:**
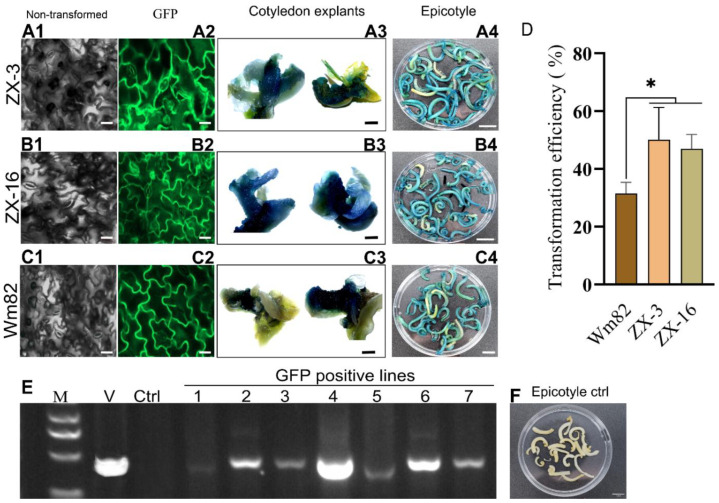
Assessment of newly developed soybean hybrid lines for *Agrobacterium*-mediated transformation. The GUS and GFP signals show different levels of transient expression in each genotype (**A1**–**A4**). Expression of GFP and GUS in ZX-3 soybean lines at different stages, (**B1**–**B4**) shows a similar pattern in ZX-16 lines, however (**C1**–**C4**) indicates GFP and GUS expression in Wm82 parental lines (**D**) Transformation efficiency of soybean hybrid lines and Wm82 control (n = 3 ± SD). (**E**) The four-sided cropped gel image results show amplification of T-DNA sections from putative transgenes (numbers from 1 to 7); here M shows a 5 kb ladder, V is a vector control, and Ctrl is a non-transformed control. The scale bar for (**A1**, **A2**, **B1**, **B2**, **C1**, and **C2**) images is 20 µm, Figure (**A3**, **B3**, and **C3**) are 1 mm, and Figure (**A4**, **B4**, **C4**, and **F**) are 1 cm. * = significant at *p* ≤ 0.05.

We performed an assessment of the increased transformation of a new soybean line resulting from the combination of susceptibility traits from the Wm82 and high flavonoid traits from the LX parents. The transformation in new lines was evaluated in explants with halved and intact cotyledons. The explants were inoculated with *A. tumefaciens* (GV3101) harboring two different vectors, the GmUbi-3XFlag-sGFP empty vector, and a reassembled GmUbi-3XFlag-35S-sGFP: GUS vector (Supplementary Figure S1). GUS expression was detected in explants after four days of co-cultivation. Here, only the inoculated epicotyl portion was subjected to GUS histochemical analysis. The GUS-staining results showed a high number of GUS-positive epicotyls in the ZX-3 lines, followed by ZX-16 and then Wm82. However, no GUS stain was observed in non-transformed control (Figure 5(A4–C4,F)). The high GUS expression level was further validated in these lines. The cotyledon explants were then cultured for shoot regeneration on SIM media supplemented with selective PGRs for two weeks. After two weeks, the explants were allowed to undergo GUS staining. In the ZX-16 and ZX-3 lines, most of the newly emerged shoots from the inoculated portion showed high GUS signals as compared to Wm82 (Figure 5(A3,B3,C3)). The identification of transgenic lines from all genotypes was achieved through the utilization of empty sGFP vectors lacking GUS assembly. The confocal microscopy results indicated that the GFP signals in the new soybean lines were markedly stronger. The count of positive GFP lines obtained from hybrid lines and Wm82 parents was utilized to ascertain the transient transformation percentage. The GFP fluorescence was observed in putative transgenic lines during the initial stages of seedling growth and subsequently in the R6 developmental stages (Figure 5(A2,B2,C2)). The leaves produce high GFP signals under 488 nm green fluorescence light, while no such signals were observed in the non-transformed control under similar fluorescence light (Figure 5(A1,B1,C1)). The GFP-positive lines were additionally validated via PCR. The T-DNA section of the vector was amplified in transgenic lines. A PCR protocol was modified to enhance band amplification. The PCR results indicated the amplification of T-DNA segments from putative transgenic lines. The vector served as a positive control, while the non-transformed line was used as a negative control. No amplification of bands was seen in the non-transformed control; however, the majority of the transgenic lines exhibited the presence of the T-DNA segment from the transformed vector (Figure 5E). GUS histochemical examination and GFP fluorescence detection revealed a high average transient transformation rate of 50.1% in the ZX-3 progenies, followed by ZX-16 at 46.9% and Wm82 at 31.4% (Figure 5D). Under some conditions, the transient transformation in the ZX-16 line exceeded that of the other lines (data not provided).

## 4. Discussion

*Agrobacterium*-mediated transformation is the simplest way to transform foreign DNA for desired changes in the plant’s genome. Nevertheless, limited transformation in certain plant species hinders the potential for rapid development of desired genotypes. In the soybean, the *Agrobacterium*-mediated transformation is more challenging compared to other crops. There are certain physiological barriers and biochemical signals that inhibit the delivery of foreign DNA, even in susceptible genotypes [24]. As previously reported, susceptible genotypes exhibit different levels of transformation, thereby decreasing the likelihood of transgene production. Exogenous biochemical and some physical methods have been used most frequently to promote soybean transformation [25]. The natural transmission of desirable traits of susceptibility among soybean cultivars has not been adequately supported by research. A singular piece of evidence regarding the quantitative inheritance of susceptibility evaluation in the soybean has been identified. Various soybean lines derived from the crossbreeding of Peking, a susceptible genotype, and Clark, a resistant genotype, exhibited distinct susceptibility patterns to *A. tumefaciens* along with enhanced agronomic traits [26]. During another event, only the evaluation of twenty-four soybean cultivars and three wild progenitors was carried out for susceptibility to *Agrobacterium*. Only three cultivars, i.e., Biloxi, Jupiter, and Peking, were found to be highly susceptible, while other progenies showed a mixed pattern of susceptibilities [27]. Our selection and research findings were comparable based on these results. To get the desired genotype, we select both genotypes with favorable alleles. Furthermore, the segregating population was not assessed until they achieved homozygosity. Because it was possible for the next generation to lose this characteristic. Among the available parents, William82 was chosen due to his high susceptibility to *A. tumefaciens*, while LX was chosen for its high anthocyanin and flavonoid accumulation. The susceptibility of soybean genotypes was investigated as one of the basic factors for high transformation [28]. Moreover, Wm82 was selected as the male parent because of its susceptibility features. This susceptibility could be easily passed from parent to offspring due to its high heritability. Conversely, the selection of high flavonoids accumulating cultivars as a female was based on two factors. Firstly, the biosynthesis of flavonoids has been investigated to be controlled by different loci, and they were negatively correlated [29]. Consequently, transferring these metabolic quantitative trait loci (mQTL) to susceptible soybean genotypes proved to be difficult. Secondly, the natural spectrum of host plant biochemicals (flavonoids) activates bacterial virulence genes and enhances host–microbe interactions during the transformation process. Consequently, we chose the LX soybean cultivar as the female parent due to its elevated flavonoid accumulation. A diverse array of polyphenolics, including flavonoids, plays a specific role in the transformation process [30]. The flavonoids are also well-known for their potential involvement in pathogen–host interaction and DNA delivery [31]. By interacting with opines, flavonoids may contribute to the porosity of the cell wall [32]. Thus, along with *vir* gene activation, flavonoids may also be effective for the safe passage of T-DNA through the cell wall [33]. The majority of these biochemicals are typically present in some susceptible soybeans in small quantities; however, for the activation of *vir* genes, a relatively high concentration is desirable [34]. The use of these phenolic compounds in media during in vitro plant propagation and transformation satisfies the need for *vir* gene activation [35]. Therefore, the transmission of traits from susceptible soybeans to high-phenolics-accumulating resistant soybean genotypes not only increased the flavonoid content but also pyramided susceptibility alleles in new progenies. As a result, this new combination of chemoattractants with susceptibility traits provided a natural way for high-efficiency *Agrobacterium* transformation. The resultant hybrid progenies were also agronomically superior. The early maturity of one of the progenies, ZX-16, was also a positive sign for high-efficiency transgene production, as early maturity was considered a sign of high *Agrobacterium* transformation in soybean [36].

## 5. Conclusions

The cost-effective nature of *Agrobacterium*-mediated transformation holds significant importance in the soybean. We presented a novel approach in the field of soybean development that involves the utilization of traditional breeding techniques to create new soybean lines that exhibit enhanced efficiency in *Agrobacterium* transformation. The result was that hybrid lines (ZX-3 and ZX-16) had more favorable allele combinations for high *Agrobacterium*-mediated transformation. Moreover, the augmentation of susceptible genotypes to *A. tumefaciens* will facilitate the soybean transformation breeding process, leading to accelerated soybean development. The significant accumulation of flavonoids and cinnamic acid in soybean hybrids is a crucial agronomic trait extending beyond mere transformation. The hybrid lines present a promising opportunity to enhance *Agrobacterium* transformation efficiency and boost the production of high-quality soybeans.

## Data Availability

The original contributions presented in the study are included in the article; further inquiries can be directed to the corresponding author.

## Supplementary Materials

The following supporting information can be downloaded at https://www.mdpi.com/article/10.3390/life14121649/s1. Supplementary Figure S1: GmUbi-3XFlag-35S-GFP empty vector was used during the studies. (A) The GmUbi-3XFlag-35S-GFP empty vector was used for transgene identification, (B) Cloning of the GUS gene in the destination vector under the expression of the GmUbi-promoter, and (C) Cloning of CaMV 35S promoter into the vector by replacing GmUbi-promoter. Supplementary Figure S2: Soybean cotyledon culture steps for transgene acquisition. (A) The imbibition of seeds was used as an explanation. (B) Following *Agrobacterium* inoculation, half-cotyledon explants were co-cultured on sterile tissue paper. (C,E) Multiple shoots were developed by the two-week SIM culture of explants. (D) Shoots were rooted on RIM for 7 days (F) Acclimatization of seedlings for one week in soil and allowed to grow further inside the greenhouse. Supplementary Figure S3: PCR results of the putative transgenic lines. The products were resolved on 1.5% agarose gel. The first seven wells, along with positive control (vector) and negative control (not transformed soybean), are given as cropped images in Figure 4 in the main manuscript file. The wells are labeled as follows: M indicates a 5 kb DNA marker; V indicates vector control; Ctrl indicates non-transformed control; and the numbers denote transgenic lines. Supplementary Table S1: Comparison of statistical significance in flavonoid abundance in soybean.
