# Supplementary material for: Generation and Assessment of Soybean (Glycine max (L.) Merr.) Hybrids for High-Efficiency Agrobacterium-Mediated Transformation
Source: Life (Basel). 2024 Dec 12;14(12):1649. doi: 10.3390/life14121649 (PMC11676979; doi:10.3390/life14121649)
Supplement: Supplementary file 1 [file life-14-01649-s001.zip › life-3341011-supplementary.pdf]

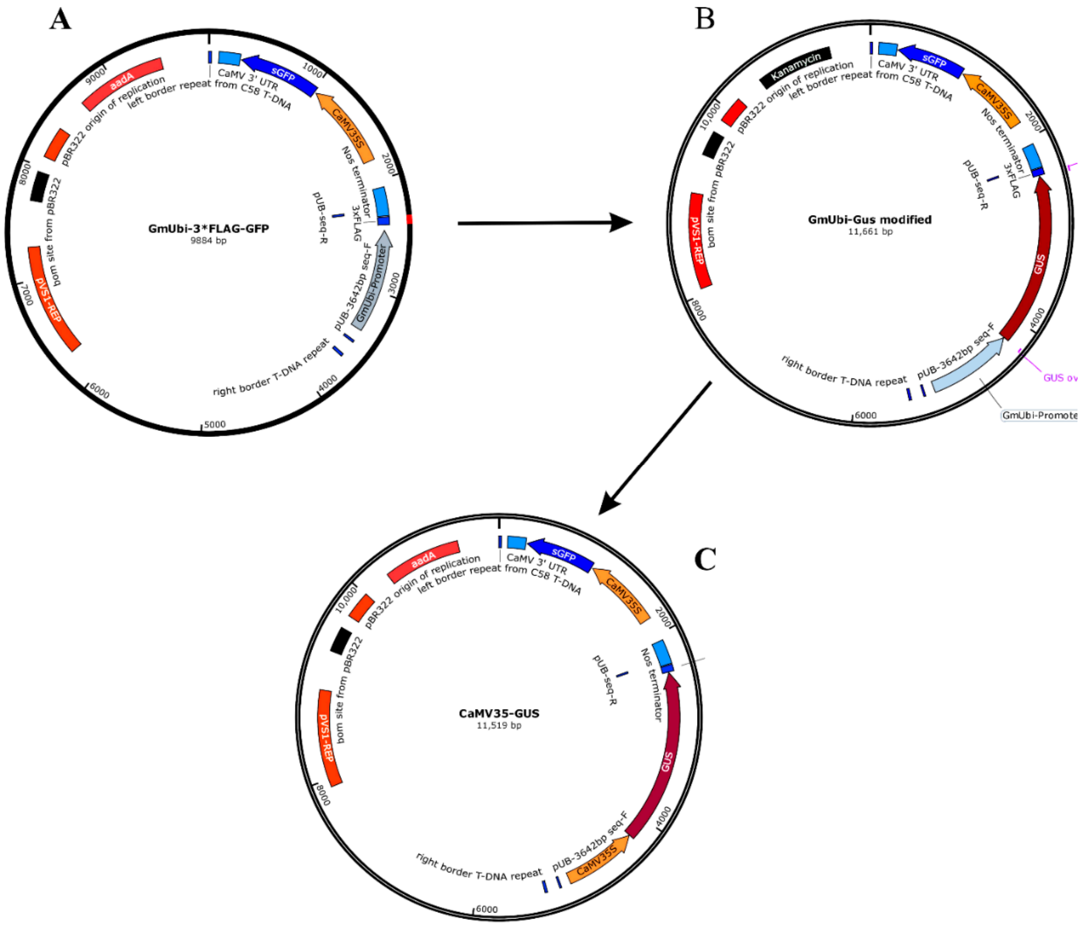

Supplementary Figure S1. GmUbi-3XFlag-35S-GFP empty vector was used during the studies. (**A**) The GmUbi-3XFlag-35S-GFP empty vector was used for transgene identification (**B**) Cloning of GUS gene in the destination vector under the expression of the GmUbi-promoter; and (**C**) Cloning of CaMV 35S promoter into the vector by replacing GmUbi-promoter.

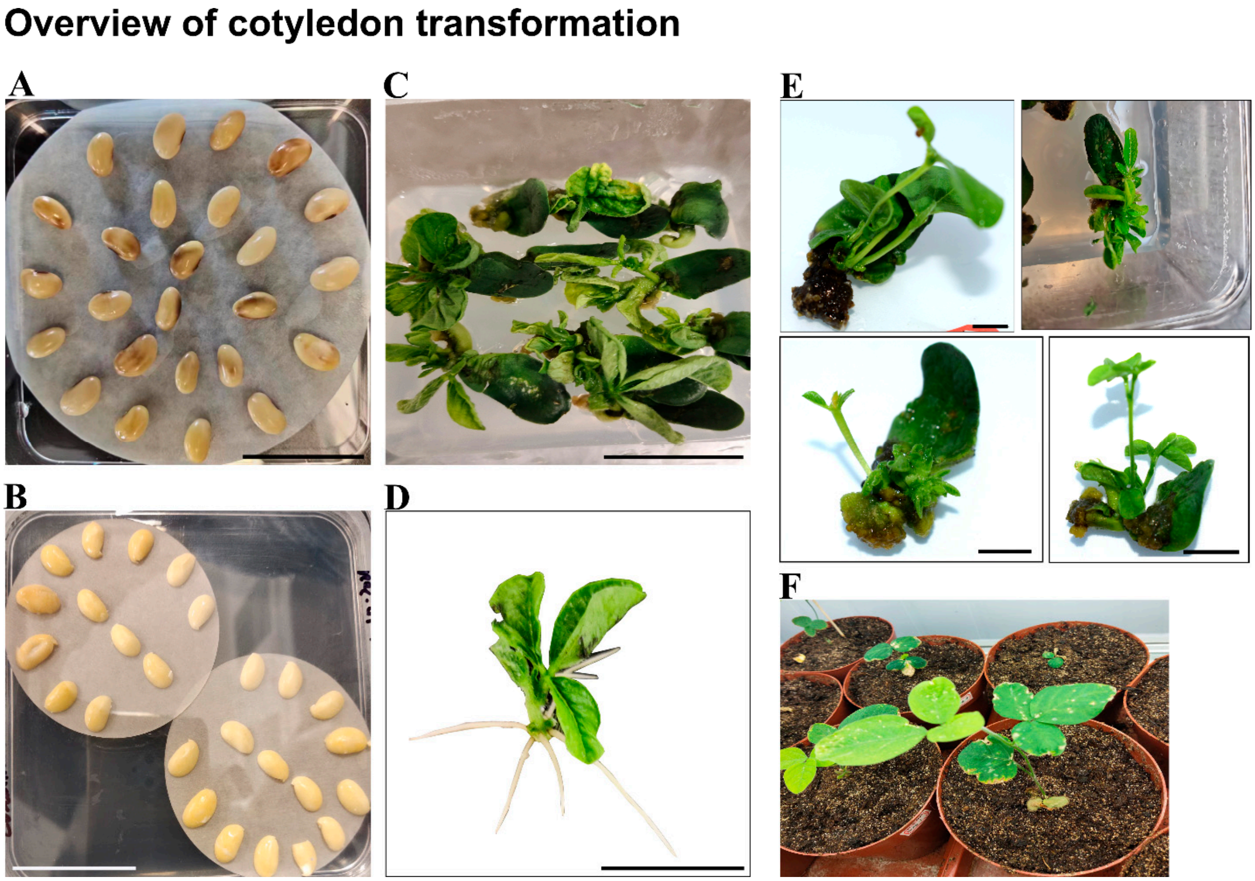

Supplementary Figure S2. Soybean cotyledon culture steps for transgene acquisition. **(A)** The imbibition of seeds that was used as an explant. **(B)** Following agrobacterium inoculation, half-cotyledon explants were co-cultured on sterile tissue paper. **(C and E)** Multiple shoots were developed by the two-week SIM culture of explants. **(D)** Shoots were rooted on RIM for 7 days **(F)** Acclimatization of seedlings for one week in soil and allowed to grow further inside the green house.

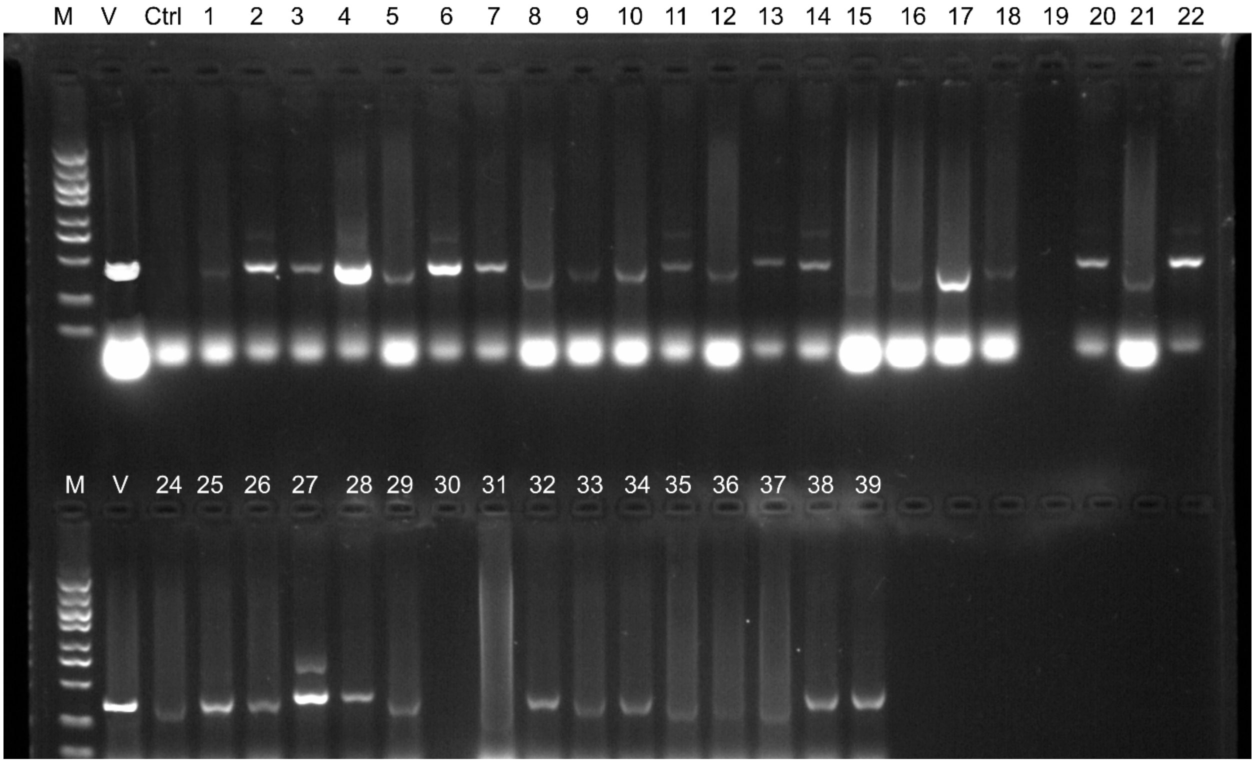

Supplementary Figure S3. PCR results of the putative transgenic lines. The products were resolved on 1.5% agarose gel. The first seven well along with positive control (vector) and negative control (not transformed soybean) has given as cropped image in Fig.4 in the main manuscript file. The wells are labeled as follows: M indicates a 5 kb DNA marker; V indicates vector control; Ctrl indicates non-transformed control; and the numbers denote transgenic lines.

Supplementary Table S1. Comparison of statistical significance in flavonoids abundance in soybean

WM82 and ZX-16							
Flavonoid	P-value	Mean of wm82	Mean of ZX-16	Difference	SE of difference	t ratio	q value
Apigenin	0.005961	3989	4247	-258.3	48.46	5.331	0.007225
Genistein	0.000232	15688	9826	5861	467.2	12.55	0.000469
Genistin	<0.000001	4178	1809	2369	46.74	50.69	0.000005
Quercitin	0.000119	142.7	565.8	-423.1	28.47	14.86	0.000362
Naringenin	0.001474	8.067	38.07	-30	3.858	7.777	0.002234
Cinnamic acid	0.008124	3003	3407	-403.9	82.67	4.886	0.008206
**WM82 and ZX-3**							
Flavonoids	P-value	Mean of wm82	Mean of ZX-3	Difference	SE of difference	t ratio	q value
Apigenin	0.000044	3989	5105	-1117	58.38	19.13	0.000022
Genistein	0.00001	15688	31730	-16042	574.4	27.93	0.00001
Genistin	0.037086	4178	4336	-158.2	51.44	3.076	0.006243
Quercetin	0.001399	142.7	70.07	72.67	9.217	7.884	0.000283
Naringenin	0.000653	8.067	22.27	-14.2	1.476	9.62	0.000165
Cinnamic acid	0.00026	3003	4352	-1349	110.7	12.19	0.000087
**ZX-3 and ZX-16**							
Flavonoids	P-value	Mean of ZX-3	Mean of ZX-16	Difference	SE of difference	t ratio	q value
Apigenin	0.000021	5105	4247	858.3	37.17	23.09	0.000007
Genistein	<0.000001	31730	9826	21903	377.5	58.03	<0.000001
Genistin	<0.000001	4336	1809	2527	39.79	63.51	<0.000001
Quercetin	0.000055	70.07	565.8	-495.8	27.36	18.12	0.000014
Naringenin	0.018452	22.27	38.07	-15.8	4.114	3.84	0.003106
Cinnamic acid	0.001959	4352	3407	945.3	131.1	7.212	0.000396
